# Longitudinal characterization reveals behavioral impairments in aged APP knock in mouse models

**DOI:** 10.1038/s41598-025-89051-8

**Published:** 2025-02-07

**Authors:** Lisa Blackmer-Raynolds, Lyndsey D. Lipson, Isabel Fraccaroli, Ian N. Krout, Jianjun Chang, Timothy Robert Sampson

**Affiliations:** 1 Department of Cell Biology, Emory University School of Medicine, Atlanta, GA USA; 2 Present Address: School of Biological Sciences, Neuroscience Undergraduate Program, Georgia Institute of Technology, Atlanta, 30332 Georgia USA

**Keywords:** Alzheimer’s disease, Cognitive behavior, Amyloid precursor protein, Animal models, Diseases of the nervous system, Alzheimer's disease, Neurodegeneration

## Abstract

APP knock-in (KI) mice serve as an exciting new model system to understand amyloid beta (Aβ) pathology, overcoming many of the limitations of previous overexpression-based model systems. The APP^SAA^ mouse model (containing the humanized APP with three familial Alzheimer’s disease mutations) and the APP^WT^ control (containing wildtype humanized APP) are the first commercially available APP KI mice within the United States. While APP^SAA^ mice have been shown to develop progressive Aβ pathology and neuroinflammation, the age at which behavioral and cognitive impairments begin to develop has yet to be described. Therefore, we performed an in-depth longitudinal study over 16 months, assessing cognition in these two strains, as well as assessments of motor function. While no cognitive deficits are observed in either genotype throughout the first year of life, 16-month-old APP^SAA^, but not APP^WT^ mice show initial signs of spatial memory decline. In addition, both genotypes display impaired motor function at the same age. Together, this data identifies a timeframe where behavioral deficits appear, providing an essential foundation for future studies using these model systems.

**Supplementary Information:**

The online version contains supplementary material available at 10.1038/s41598-025-89051-8.

## Introduction

Establishing and characterizing animal models that accurately recapitulate pathological and behavioral outcomes of human disease remains an essential component—and limiting factor—to understanding disease etiology and identifying novel treatment targets. Rodent models of amyloid beta (Aβ) pathology—a key hallmark of Alzheimer’s disease (AD), as well as a prominent co-pathology in Parkinson’s disease (PD), Lewy body dementia (LBD), and other amyloid diseases—have been utilized for decades^1,2^. These models have provided important insights into the role of Aβ in neurodegeneration and other physiological processes. Early rodent models of Aβ pathology were first developed by overexpressing the human amyloid precursor protein (APP) gene containing highly amyloidogenic mutations associated with familial AD (FAD) in humans^3–5^. The majority of amyloid transgenic mouse models in use today follow a similar, overt overexpression approach, driving high expression of various combinations of transgenes of APP, presenilin, tau, and alpha-synuclein^1,6^. However, there is increasing recognition that these overexpression approaches also have a number of important limitations. These include random gene integration, ectopic expression, overrepresentation of specific splice variants, non-physiological drivers of protein expression, and an inability to assess transcriptional regulatory impacts on the gene of interest, all of which confound experimental results^7^.

In response to these limitations, various knock-in (KI) models of amyloid pathology have been created in which the murine gene of interest is knocked-out and replaced with a humanized transgene either at the locus itself or in *trans*^1,8–15^. These KI models overcome many of the limitations of overexpression-based models because the transgenes are expressed closer to physiological levels and are driven by native promotors, generating much excitement within the research community^7^. The first commercially available KI model of Aβ pathology in the United States, the APP^SAA^ mouse, carries humanized APP with three mutations associated with FAD (the Swedish, Arctic, and Austrian mutations)^15^. Molecular pathologies of the APP^SAA^ mouse have been extensively characterized and demonstrate the development of amyloid and tau pathology, neurodegeneration, neuroinflammation, and neurovascular deficits that recapitulates elements of human disease^15–18^. In addition, studies have demonstrated hyperactivity behavior at 18 months^15^, cognitive impairments on contextual and cued fear conditioning tests at 12–13 months^17^ and object recognition and radial arm maze deficits at 7.5 months^18^. However, it is currently unknown when these behavioral impairments begin to develop and how they progress as the animal ages. To address this, here we performed an in-depth, longitudinal characterization of various aspects of rodent behavior known to be impacted by amyloid pathology including anxiety-like behaviors, learning and memory, as well as motor function in APP^SAA^ mice. These behavioral assessments were performed side-by-side in APP^WT^ mice, another under-characterized APP KI model. APP^WT^ mice encode humanized, wild-type APP without any FAD mutations and have significantly less Aβ accumulation than APP^SAA^ mice^17^. While recommended as a control animal for the APP^SAA^ mice, this humanized KI mouse model also has the potential to develop behavioral deficits that have yet to be characterized.

We initially predicted that both mouse strains would display progressive behavioral impairments due to the presence of the human APP gene, with the APP^SAA^ mice displaying exacerbated impairments compared to mice harboring the wildtype human gene. While we observed human Aβ accumulation in the brains of both genotypes, which was significantly higher in the APP^SAA^ mice, neither strain developed progressive cognitive or motor deficits within the first year of life. However, APP^SAA^ mice were found to display significant learning and memory deficits on the object location and Barnes maze tests at 16 months of age. In addition, both APP KI genotypes displayed motor impairments at 16 months. The age between 12 and 16 months therefore represents a potential tipping point whereby APP KI mice display age- and genotype-dependent cognitive impairment, an essential parameter for the study of disease modifying factors of amyloid pathology.

## Materials and methods

### Animal husbandry

Female and male APP^SAA^ KI (B6.Cg-Apptm1.1Dnli/J Strain #:034711) and APP^WT^ KI (B6.Cg-Appem1Adiuj/J Strain #:033013) mice were acquired from Jackson Labs through a kind gift by Dr. Srikant Rangaraju (Emory University) and maintained as homozygotes. The APP^SAA^ model was created by humanizing the Aβ region of the APP gene using R684H, F681Y, and G676R mutations, then adding 3 additional FAD mutations: the KM670/671NL (Swedish) mutation in exon 16 as well as the E693G (Arctic) and T714I (Austrian) mutations in exon 17^15^. The APP^WT^ mouse contains a humanized Aß1–42 region (G601R, F606Y, R609H in the mouse gene, corresponding to amino acid positions 676, 681, 684 in the human *APP* locus), but no additional FAD mutations. Both genotypes were maintained on a C57BL/6J background. Mice were genotyped by PCR using primers and conditions per the vendor (Jackson Labs). Mice were housed with 2–5 sex and age matched cage mates of the same genotype within unenriched microisolator cages in a central, specific pathogen-free vivarium on ventilated racks, with food (LabDiet: 5001) and water provided *ad libitum* and a 12:12 h light-dark cycle. All animal experiments were performed during the animal’s light cycle. Animal husbandry and experiments were performed in accordance with relevant guidelines and regulations, approved by the Institutional Animal Care and Use Committee of Emory University (PROTO201900056), and reported in accordance with ARRIVE guidelines.

### Overview of cognitive behavioral testing

Since, APP^SAA^ mice have been shown to develop pathology starting at 4 months of age^15^ we began longitudinal cognitive testing monthly at 4, 5, and 6 months of age to see if early pathology would impact behavior, however, when no cognitive impairments were observed the duration of testing was spread out and repeated again on the same animals at 12 months, when previous reports^17^ had shown deficits. Finally, to test an even later age and mitigate any potential confounds of repeat testing, mice were also tested cross sectionally at 2–3 months old and 16 months old. Each genotype and age included 9–13 mice with roughly equal numbers of males and females as indicated in the associated Supplementary Information files. At each timepoint, the following tests were performed in order: open field test, object location test, Y maze, and Barnes maze. Before the start of any test, mice were habituated to the testing room in their home cage for 1 h. All behavioral tracking and analysis was performed using EthoVision XT software (version 16.0.1538; Noldus Information Technology, Wageningen, the Netherlands; https://www.noldus.com/ethovision-xt) and the testing arenas/objects were cleaned between trials with 70% ethanol to eliminate olfactory cues.

#### Open field test (OFT)

During the OFT, mice were placed in a 45 cm square open field box for 10 min while distance traveled and time spent in the center was recorded as measures of motor and anxiety-like behavior.

#### Object location test (OLT)

24 h post OFT, the OLT was run as described step-by-step in^19^ to assess short-term spatial memory. Briefly, mice were placed in an open field with landmarks on 3 out of the four walls to allow for spatial orientation. During the initial study phase, mice were placed in the box with two identical copies of an object and allowed to explore freely for 10 min. The mice underwent a 10-minute retention delay in their home cage before being returned to the field for 5 min, with one of the two objects placed in a new location. Object exploration was considered time spent with the mouse’s nose within 2 cm of an object. Exploration ratio = moved object exploration/total object exploration. An exploration ratio significantly above chance levels of 0.5 is indicative of intact memory as mice generally seek out the moved object.

#### Y-maze

Mice were then tested on the Y-maze to evaluate spatial working memory, as described in detail in^20^. Mice were allowed to freely explore a Y shaped maze for 8 min while entries into each arm were recorded. Percent alternation = total alternations (consecutive entries into 3 arms before repeating any arms) / maximum possible alternations (total entries minus 2). Mice with intact working memory display higher percent alternation as mice prefer to explore previously unvisited areas.

#### Barnes maze

Barnes maze testing adapted from^21^, and described in detailed in^22^ was used to assess spatial learning and memory. Testing occurred on a 92 cm diameter, 20 hole, Barnes maze (MazeEngineers) over a 6-day period, with one habituation trial, five 3-minute training trials, and a 72-hour probe trial. During habituation, mice were placed on the maze with bright lights and white noise (66–70 dB) playing for 20 s before being gently guided to an escape hole leading to a dark box. Upon entry to the box, the white noise was turned off and the mice were allowed to rest in the escape box for 2 min. During each training trial, mice were once again placed on the maze with white noise playing and allowed to explore for up to 3 min. Upon entry into the escape box, the noise was turned off and mice were allowed to rest for 1 min. Mice that did not enter the escape box after 3 min were guided to the proper hole. 72 h after the final training trial, mice were returned to the maze with the escape box removed and their behavior was observed for a 90 s probe trial. Primary latency (time until the mouse first checked the escape hole) was recorded for each trial. During repeat testing, the location of the escape hole was shifted 90 degrees to prevent mice from remembering the previous location of the escape hole. The extra-maze cues remained the same throughout all testing sessions.

### Motor behavior assessments

Since motor impairments typically develop later in the progression of amyloid pathology^23^, motor function testing was performed cross sectionally at 3, 12, and 16 months of age as follows.

#### Adhesive removal

The adhesive removal test was performed as described in ^24^, by placing an adhesive sticker onto the mouse’s nose and quantifying the time needed to remove the sticker across three consecutive trials. This test provides insight into both sensory perception and fine motor skills.

#### Wirehang

The wirehang test was performed as described in^25^ to evaluate muscle strength and function. Briefly, mice were placed on a 43 cm square wire screen (made up of 12 mm squares of 1 mm diameter wire), turned upside down, and the time the mouse held onto the screen was recorded and averaged across three consecutive trials.

#### Hindlimb rigidity

Hindlimb scoring was performed on a scale of 0–3 based on extent of hindlimb clasping adapted from^26^ and described in^27^ on two separate days by two independent scorers. Greater hindlimb clasping is seen in mice with motor deficits and is indicative of neurodegenerative disease progression.

### Tissue collection and Aβ quantification

At 12 months of age, mice were humanely euthanized under isoflurane anesthesia and perfused with PBS. Aβ levels were quantified in the hippocampus since this is a region known to have the highest Aβ burden in APP^SAA^ mice^17^. Hippocampal tissue was dissected, flash frozen, and protein extracted using a two-part fractionation protocol^28^. Tris soluble (1 M Tris HCL, 0.5 M MgCl2 and 0.1 M EDTA- pH 7.8) and Triton soluble (1% Triton-X100) fractions were run on the Meso Scale Discovery V-PLEX human Aβ peptide kit (K15200E) according to the manufacture’s guidelines. The resulting protein values were normalized to frozen tissue weight (rather than total protein concentration) since the total protein volume of the less soluble fractions may be influenced by the amount of insoluble Aβ present in the tissue.

### Statistical analysis

As indicated in the figure legends, data are expressed as mean ± SEM. Statistical tests were performed using GraphPad Prism 8. Longitudinal data was analyzed using repeated measures one- or two-way ANOVAs and cross-sectional data was measured using standard ANOVAs. The OLT outcome was also measured using a one sample t test compared to 0.5 chance level and Barnes maze training trials were analyzed by comparing the area under the curve (AUC) from all five training trials. All raw numerical data and statistical outputs are included with the manuscript as Supplementary Information files.

## Results

### Limited cognitive impairment is observed longitudinally through twelve months of age

**Fig. 1:**
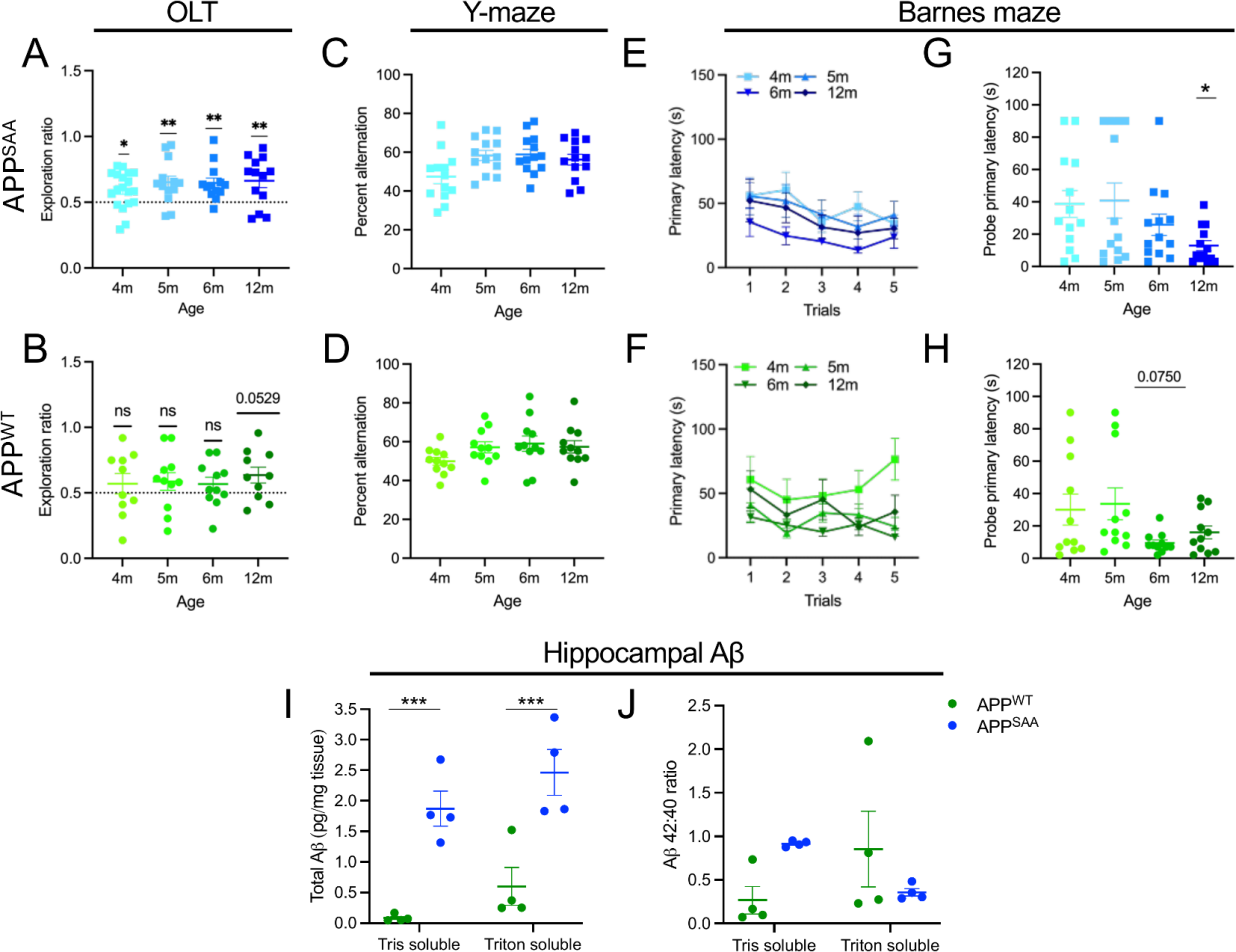
APP-KI mice do not show progressive cognitive impairment through 12 months of age. Male and female APP^SAA^ (**a**,**c**,**e**,**g**) and APP^WT^ (**b**,**d**,**f**,**h**) mice were tested longitudinally from 4 through 12 month (m) of age, at the indicated time points across a battery of cognitive behavior tests. (**a**,**b**) Exploration ratio in the object location test (OLT). (**c**,**d)** Percent alternation in the Y-maze. (**e**,**f**) Barnes maze performance during longitudinal training period and (**g**,**h**) 72 h probe trial. (**i**,**j**) Tris soluble and Triton soluble hippocampal Aß analyzed by multiplex ELISA. (**i**) Total Aβ including Aβ38, Aβ40, and Aβ42 (**j**) Aβ 42:40 ratio. A significant genotype by solubility interaction effect was observed in Aβ 42:40 ratio (*p* = 0.0297). Points represent individuals (excluding (**e**,**f**) where points represent the group mean), bars represent the mean ± SEM. *n* = 10–11 APP^WT^ and 13 APP^SAA^ for (**a-h**) and *n* = 4 for (**i-j**). Data analyzed by one sample t test compared to 0.5 chance level for (**a**,**b**) repeated measures ANOVA and Dunnett’s multiple comparisons test to compare each age to 4 m timepoint (**c-h**) and 2-way ANOVA with Fisher’s LSD post-hoc tests I-J. *p ≤ 0.05; **p ≤ 0.01; ***p ≤ 0.001.

In order to test the hallmark, progressive, cognitive impairments observed in amyloid diseases, and other amyloid mouse models, we examined both APP KI genotypes across a battery of behavioral tests through aging. Mice were first evaluated longitudinally starting at 4 months of age (when pathology begins to develop in the APP^SAA^ mice^15,16^) to assess whether cognitive decline develops within the first year of life. However, throughout the first 12 months of age, neither genotype displayed progressive cognitive impairment on any of the behavioral tests (Fig. 1). In the object location test (OLT), the APP^SAA^ mice maintained a consistent discrimination of the object in a novel location—represented by an exploration ratio significantly above 0.5 chance levels with no significant difference in discrimination ratio between ages (Fig. 1a). However, the APP^WT^ genotype consistently performed no better than chance, with a trend towards novelty preference (*p* = 0.053) only appearing at 12 months (Fig. 1b). This is despite no significant difference in total object exploration between the two genotypes at any timepoint, but a significant overall decline in object exploration with repeat testing in the APP^WT^ mice at 12 months (Supplementary Fig. S1a).

Similarly, in the Y-maze test of spatial working memory, we observed no progressive loss of working memory (indicated by a decline in percent alternation) in either genotype through 12 months of age (Fig. 1c, d). Of note, however, there was a significant negative correlation between percent alternation and the total number of arm entries for both genotypes, especially the APP^SAA^ mice, suggesting the animal’s activity level in this specific test may confound an interpretation of their cognitive performance (Supplementary Fig. S1b, c). To determine general locomotor activity and evaluate potential anxiety-like behaviors that could impact cognitive outcomes, the open field test (OFT) was also performed. While we observed a gradual decrease in distance traveled over time—a common finding as animals become habituated to the repetitive testing environment^29^—differences in distance traveled between the two genotypes only appeared at 4 months of age (Supplementary Fig. S1d). In addition, 4-month-old APP^SAA^ mice spent more time in the center during the open field compared to APP^WT^ at the same age suggesting potential genotype-dependent effects in anxiety-like behavior that dissipates with age (Supplementary Fig. S1e).

In the Barnes maze, both genotypes were able to quickly learn the location of the goal box even at 12 months of age (Fig. 1e, f). In fact, mice in both genotypes showed a median primary latency (time to first identify the target hole) below 30 s (as is typically seen after successful Barnes maze training^21^) within the first two training trials, suggesting that the mice learned the location of the goal box during habituation and the first training trail. While primary latency did not significantly decrease over subsequent training sessions, this is likely due to a plateau effect rather than a learning deficit. While repeat testing could theoretically decrease initial primary latency measures if mice remember the location of the escape hole from the previous test, no significant difference in primary latency was observed during any training trial, suggesting that the tests were far enough apart to not require reversal learning (Fig. 1e, f). Following a 72 h retention interval, all ages and genotypes showed similar or improved primary latency, suggesting no progressive loss of memory over time (Fig. 1g, h).

To confirm the presence of increased human amyloid beta (Aβ) in each KI model, we performed detergent fractionation of hippocampal tissue lysates at 12 months of age. In line with prior reports^15,17^, we observed the presence of both Tris soluble and detergent soluble Aβ by ELISA, with generally increased levels in the APP^SAA^ genotype (Fig. 1i, j). While some homogenization bias may exist between samples, due to normalization by total tissue weight, we expect this variability to be equal across these groups during processing. Given the behavioral outcomes, we conclude that the presence of amyloid burden at this age is not sufficient to induce cognitive impairment in these hallmark behaviors.

### APP^SAA^ mice display cognitive impairment at 16 months of age

**Fig. 2:**
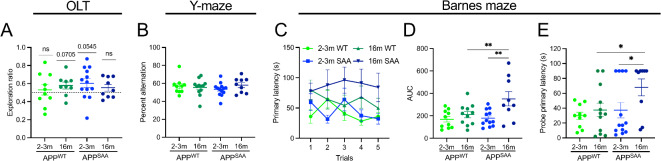
APP^SAA^ but not APP^WT^ mice show spatial learning and memory deficits at 16 months of age. Young (2–3-month-old) and 16-month-old male and female APP^SAA^ and APP^WT^ mice were tested cross sectionally on a range of cognitive tests. (**a**) Exploration ratio in the object location test (OLT). (**b**) Percent alternation in the Y-maze. (**c-e**) Barnes maze performance during longitudinal training period showing both (**c**) primary latency and (**d**) area under the curve (AUC). (**e**) 72 h probe trial primary latency. Points represent individuals (excluding **c**, where points represent the group mean), bars represent the mean and SEM. *n* = 9–12 APP^WT^ and 9–13 APP^SAA^. Data analyzed by one sample t test compared to 0.5 chance level for (**a**), 2-way ANOVA with Fisher’s LSD post hoc test for (**b**,**d** and **e**). *p ≤ 0.05; **p ≤ 0.01.

While we did not observe cognitive impairment through 12 months of age, similar models can begin behavioral deficits later in life^12^. We therefore performed a cross-sectional analysis between independent cohorts of young, 2–3-month-old APP^WT^ and APP^SAA^ mice compared to individuals aged through 16 months-old (Fig. 2). While 2–3 month APP^SAA^ mice showed a clear trend towards a novelty preference on the OLT (*p* = 0.054), 16 month APP^SAA^ mice had a novelty preference score that was no different than chance levels (despite spending similar time exploring the objects, Supplementary Fig, S2a), suggesting that they were no longer able to remember which object had been moved (Fig. 2a). Between groups analysis did not reach significance, however, likely due to lower exploration ratios in the young mice than had been previously seen in Fig. 1. In addition, there was still no significant difference in percent alternation on the Y maze based on age or genotype (and no correlation between percent alternation and number of entries, Supplementary Fig. S2b, c), suggesting that all mice have similar working memory (Fig. 2B), irrespective of age or genotype. Finally, in the Barnes maze, 16-month APP^SAA^ mice showed a significant learning and memory deficit. Compared to both young APP^SAA^ and 16 month APP^WT^ mice, 16 month APP^SAA^ mice never were able to learn the escape hole location, with a significantly higher training AUC than the young mice of either genotype (Fig. 2c, d). Similarly, 16-month APP^SAA^ mice took significantly longer to find the escape hole during the probe trial than the other groups (Fig. 2e). While the mixed-sex cohorts used in the present study are not sufficiently powered to evaluate sex differences, 2–3 month male APP^SAA^, appear to have worsened cognitive impairment compared to females, highlighting the need for future studies to investigate sex differences in these models (Supplementary Fig. S2d). Together, these results suggest that APP^SAA^ but not APP^WT^ mice display cognitive deficits at 16 months old.

Since motor impairments are associated with the development of amyloid pathology, motor testing was also performed within both APP KI genotypes. Mice were tested cross-sectionally, at 3, 12, and 16 months. While both genotypes showed intact sensorimotor function during the nasal adhesive removal test and motor function on the wire hang test and at 3 and 12 months, 16-month-old mice of both genotypes show significant impairments in these behaviors (Supplementary Fig. S2e, f). In addition, APP^SAA^ mice fell from the wirehang test more quickly than the APP^WT^ mice at 16 months, suggesting a greater motor impairment in this genotype (Supplementary Fig. S2f), even when normalized by body weight (Supplementary Fig. S2g). Even at 16 months neither genotype displayed limb rigidity on the hind limb test (Supplementary Fig. S2h). APP^SAA^ mice were heavier at 16-months of age than APP^WT^ animals (Supplementary Fig. S2i, j), which may contribute to performance in some of these tests. In the OFT, APP^WT^ mice traveled further than the APP^SAA^ mice, particularly at 3-month of age (Supplementary Fig. S2k). In addition, there was a significant reduction in the total distance traveled between the young 2–3-month APP^WT^ mice and the 16-month APP^WT^ mice (Supplementary Fig. S2k). There was, however, no difference in time spent in center across genotypes or ages, suggesting that anxiety-like behavior remains stable up to 16 months (Supplementary Fig. S2l).

## Discussion

KI models, such as the APP-KI strains used in this study, represent an important tool to understand etiological mechanisms that underlie amyloid pathologies and therapeutic interventions aimed at their clearance. By removing the presence of the murine amyloid ortholog, potential confounding interactions between the human and murine amyloids are avoided. Transcriptional control by the native human promoter in these models further allows a clearer understanding of how amyloid proteins respond to disease-relevant insults, such as immune modulation, metabolic input, or environmental exposures. In order to effectively use these models, however, it is critical to evaluate baseline pathologies and behaviors to have a foundation to explore such perturbations.

The two APP KI genotypes used in this study are emerging and recently described model systems that currently lack complete characterization. While prior studies have characterized the longitudinal development of amyloid pathology and neuroinflammation^15,16^ and demonstrated behavioral abnormalities late in life in the APP^SAA^ mice^15,17,18^, to our knowledge, there is no published dataset on the age-related development and progression of cognitive behaviors of either KI genotype. We therefore set out to identify a behavioral “tipping point,” an age window where these genotypes show cognitive impairment or where the more pathogenic APP^SAA^ genotype separate behaviorally from the APP^WT^ genotype. Such a timepoint is important for timing potential interventions that seek to accelerate or diminish disease outcomes, directly test etiological contributions, or evaluate therapeutic interventions.

APP^SAA^ mice have been reported to display progressive amyloid pathology and neuroinflammation starting at 4 months of age^15,16^. We therefore hypothesized that these mice would display cognitive impairment as pathology progressed. However, despite assessing behaviors longitudinally for 12 months and confirming the presence of human Aβ accumulation in both mouse models, we were unable to detect progressive cognitive impairment in either genotype within the first year. This stands in contrast to previously published papers showing deficits on the radial arm maze and object recognition test in APP^SAA^ mice at 7.5 months^18^ and in cued fear conditioning in APP^SAA^ and contextual fear conditioning tests in both genotypes at 12-13.5 months old compared to WT C57BL/6J controls^17^. This discrepancy is likely due to differences in the cognitive tests performed and overall experimental design.

While we were unable to detect significant age-related cognitive decline in the first year of life, each genotype displayed significant behavioral impairments at 16 months of age. Both genotypes showed significant motor deficits on the wire hang and adhesive removal test, suggesting that the human APP KI is sufficient to modulate motor outcomes at this age, regardless of the presence of FAD mutations. In addition, 16-month-old APP^SAA^, but not APP^WT^ mice displayed spatial memory and learning deficits on the OLT and Barnes maze tests, highlighting an age by genotype interaction where the more susceptible APP^SAA^ mice begin to show cognitive decline sometime between 12 and 16 months of age. Since these tests were performed cross sectionally rather than longitudinally, it is difficult to directly compare these results to those of previous ages. Nevertheless, these results suggest that the time period between 12 and 16 months of age likely represents the range where age- and genotype-dependent behavioral deficits associated with amyloid disease manifest in these mice.

Of note, the APP^WT^ mice performed at chance levels on the OLT at all timepoints, suggesting possible behavioral abnormalities even in young mice. While the APP^WT^ mouse is considered a control for the APP^SAA^ genotype, they are themselves a KI model and therefore may display their own behavioral deficits caused by the insertion of the wildtype humanized APP gene. However, lack of observable cognitive deficits on the other cognitive tests, suggests that this deficit is either very specific to the type of short-term object location memory tested, or is due to a confounding variable such as differences in motivation, hyperactivity, or visual acuity. Indeed, 2–3-month-old APP^WT^ mice display increased distance traveled on the OFT compared to APP^SAA^ mice. While this trend remains at 16 months of age, there is a significant decrease in distance traveled in the APP^WT^ mice with age, perhaps explaining why the older APP^WT^ mice have OLT discrimination ratios that are almost significantly above chance levels. Together, these results suggest that OLT may not be a suitable test for evaluating cognitive decline in APP^WT^ mice. Use of cognitive tests that are not shaped by hyperactivity would allow for a more accurate evaluation of age-related cognitive performance in this model.

Within established APP KI models, there is considerable variability in the age of cognitive decline depending on the presence of specific FAD mutations. For example, the APP^NL−G−F^ model (containing the Swedish, Iberian, and Artic mutations) has been shown to display cognitive impairment on the Y maze starting at 6 months of age, but the APP^NL−F^ model (containing only the Swedish and Iberian mutations) does not show impairment in this test until 18 months^12^. While the APP^SAA^ model contains the Swedish and Artic mutations like the APP^NL−G−F^ model, the presence of the Austrian mutation, rather than the Iberian mutation may impact the age of onset for cognitive decline, which we observe at 16 months of age. While cognitive decline was not observed in APP^WT^ mice at 16 months of age, it is possible that cognitive impairment could appear in this model at even later ages or with more sensitive tests. For example, contextual fear conditioning deficits have been reported in 12-13.5 month old APP^WT^ mice^17^. Wildtype C57BL/6J mice are reported to display cognitive and motor impairments at 18–20 months of age, so it is possible that the APP^WT^ mice will not begin to show more striking cognitive deficits in other behavioral paradigms until this age^30–32^.

### Limitations of the study

While the present study provides important insight into age-related behavioral changes in APP^SAA^ and APP^WT^ mice, there are several limitations that should be taken into consideration. While comparing within genotypes of different ages rather than between genotypes allowed us to identify the age-range in which each genotype displays behavioral abnormalities, this may be less sensitive to subtle genotype-dependent differences in cognitive performance compared to purely wild-type mice. Since mice were not tested between the ages of 12 and 16 months, it is difficult to pinpoint the exact age when behavioral abnormalities arise as they could appear anytime within this window. Similarly, our choice of longitudinal testing, with a repeated measures design, may also have masked more subtle cognitive deficits at earlier ages, as mice may have an easier time with a task that they have performed before. However, even at the youngest ages and within the APP^WT^ genotype where no cognitive impairment is expected, repeat testing did not result in significantly improved performance, suggesting that the effects of repeat testing were likely minimal.

We utilized mixed-sex cohorts throughout our experiment, however this study was not designed to identify sex differences or sex-by-age interactions. It is therefore possible that behavioral differences in a single sex may be driving or masking the earlier development of behavioral deficits within these model systems. While underpowered, we did observe a subtle exacerbation of cognitive impairment on the Barnes maze in 2–3 month male APP^SAA^ mice compared to females, suggesting that sex differences may well be present within these mice. However, this result stands in contrast to a recently published study showing contextual and cued fear conditioning deficits in female, but not male mice at 12-13.5 months of age^17^, highlighting the need for future studies to further evaluate sex differences in these models. While our study is unable to evaluate these differences, it provides the time frame that future studies can utilize to evaluate these, and other experimental questions, in each model system.

## Conclusions

No single AD mouse model can fully recapitulate the human condition; however, when properly characterized, model systems are essential tools to address specific biological and translational questions. The present study provides a longitudinal characterization of the behavioral phenotypes of APP^SAA^ and APP^WT^ mice in a mixed-sex cohort over the course of 16 months, providing foundational behavioral data to inform future work. We have identified an age at which APP^SAA^ mice show significant cognitive decline compared to APP^WT^ mice, providing a critical behavioral window for studies designed to limit or exacerbate behavioral defects. Further, we have identified age-related motor dysfunctions in both mouse models that will be important to consider both in the interpretation of cognitive behaviors and in the study of co-morbidities of amyloid diseases. Future studies into how various insults shape pathological and behavioral outcomes in these relevant Aβ-dependent mouse models will provide substantial insights into how various factors interact to promote disease.

## Electronic supplementary material

Below is the link to the electronic supplementary material.

Supplementary Material 1

Supplementary Material 2

Supplementary Material 3

## Data Availability

All data generated or analysed during this study are included in this published article and its supplementary information files.
