# Supplementary material for: Longitudinal characterization reveals behavioral impairments in aged APP knock in mouse models
Source: Sci Rep. 2025 Feb 7;15:4631. doi: 10.1038/s41598-025-89051-8 (PMC11805898; doi:10.1038/s41598-025-89051-8)
Supplement: Supplementary file 2 — Supplementary Material 2 [file 41598_2025_89051_MOESM2_ESM.pdf]

Longitudinal characterization reveals behavioral impairments in aged APP Knock in mouse models.

Lisa Blackmer-Raynolds and Lyndsey D. Lipson et al.

Supplementary Materials

-Supplementary Figures 1 and 2

Supplementary Figure 1.

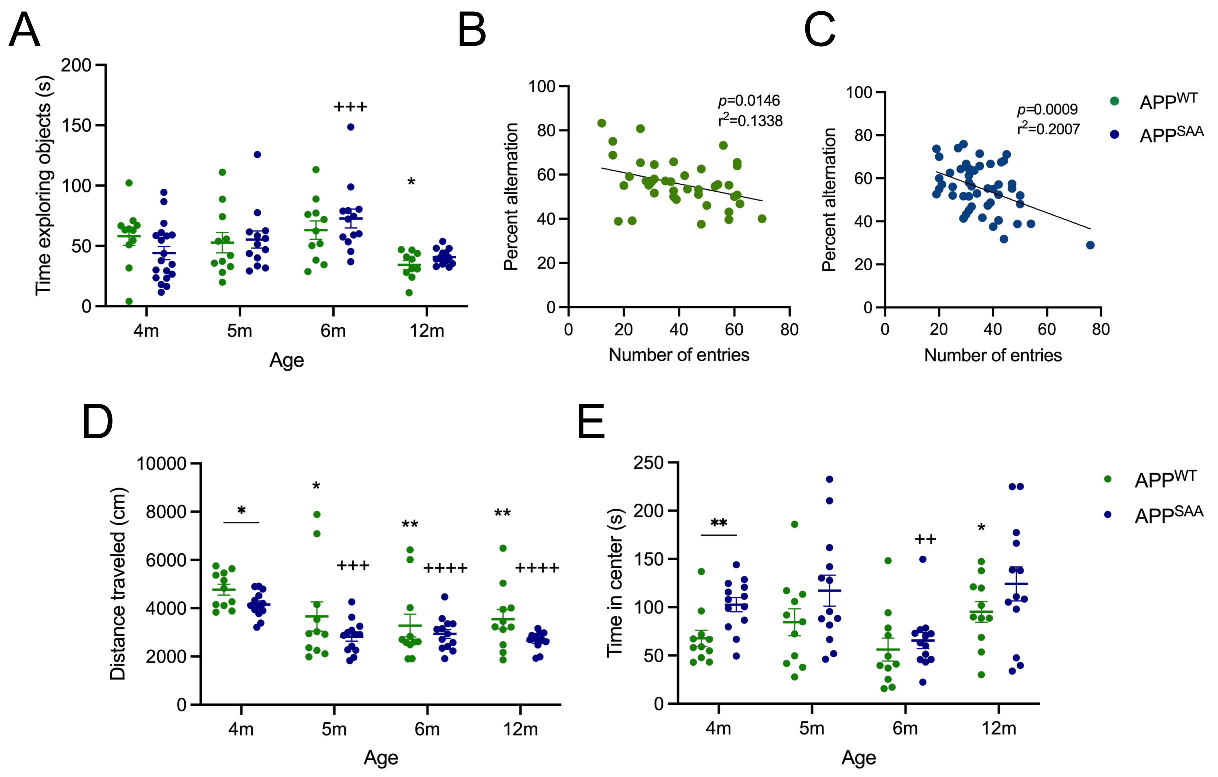

Supplementary Figure 1. Limited behavioral abnormalities appear in APP-KI mice through 12 months of age. **A)** Total object exploration during OLT. **B, C)** Correlation between number of entries and percent alternation during the Y maze for APP^{WT} **(B)** and APP^{SAA} **(C)** mice respectively. **D, E)** Male and female APP^{SAA} and APP^{WT} mice were tested longitudinally on the open field test from 4-12 months (m) of age. **D)** Total distance traveled and **E)** time spent in center during 10-minute testing period. Points represent individuals, bars represent the mean and SEM. n= 10-11 APP^{WT} and 13 APP^{SAA} for **A, D, & E**. Data analyzed with a 2-way ANOVA with Fisher's LSD post hoc test for **A, D, & E** and simple linear regression for **B-C.** *$p \leq 0.05$; **$p \leq 0.01$; ***$p \leq 0.001$ compared to 4m APP^{WT} mice. +$p \leq 0.05$; ++$p \leq 0.01$;+++$p \leq 0.001$, ++++$p \leq 0.0001$ compared to 4m APP^{SAA}.

Supplementary Figure 2.

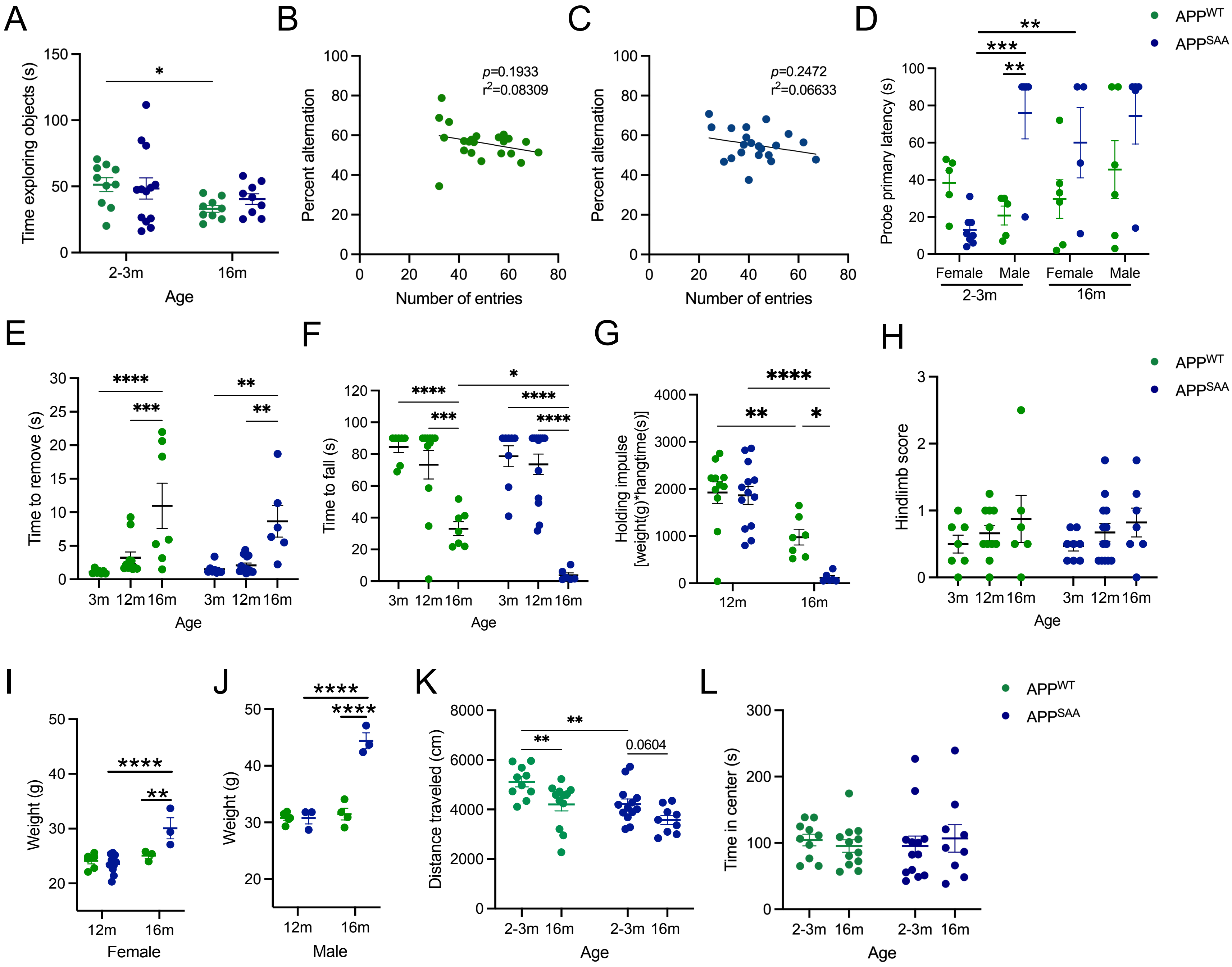

Supplementary Figure 2. APP-KI mice show age related motor impairments regardless of genotype. Male and female APP^{SAA} and APP^{WT} mice were tested cross sectionally at 2-3, 12, and 16 months (m) of age. **A)** Total object exploration during OLT. **B,C)** Correlation between number of entries and percent alternation during the Y maze for APP^{WT} **(B)** and APP^{SAA} **(C). D)** Primary latency in the Barnes maze, from Figure 2E, separated by biological sex. **E)** Time to remove a nasal adhesive in sticker removal test. **F)** Time to fall on the wire hang test. **G)** Hanging Impulse score. **H)** Hindlimb rigidity score. **I, J)** Body weight of female **(I)** and male **(J)** mice at 12 and 16m of age. **K)** Distance traveled and **L)** time spent in center during open field test. Points represent individuals, bars represent the mean and SEM. n= 7-11 APP^{WT} and 8-13 APP^{SAA}. Data analyzed by 2-way ANOVA with Fisher's LSD post-hoc tests comparing each genotype and age (or sex) for **A, D-K** and simple linear regression for **B-C**. *$p \leq 0.05$; **$p \leq 0.01$; ***$p \leq 0.001$; ****$p \leq 0.0001$.
